# Screening for delirium within intensive care units in scotland: a national survey

**DOI:** 10.1186/2197-425X-3-S1-A727

**Published:** 2015-10-01

**Authors:** AW McGuire, LG Young, JAG Davidson

**Affiliations:** Victoria Infirmary/ NHS Greater Glasgow & Clyde, Intensive Care, Glasgow, United Kingdom

## Introduction

Delirium is defined by the Diagnostic and Statistical Manual of Mental Disorders (DSM-IV) as a disturbance in consciousness and alteration in cognition over a short time period. It tends to fluctuate throughout the day and is evident from the clinical history, examination or investigations that delirium is as a result of a medical condition, drug withdrawal or intoxication. All of the above factors need to be present for a diagnosis of delirium to be made.¹

In the UK around 65% of unwell ventilated patients in intensive care experience delirium.² This can be challenging to diagnose in the severely unwell patient. Delirium is associated with increased morbidity and mortality, increased length of hospital stay and long term disability and dementia.² The American College of Critical Care Medicine and Intensive Care society recommend daily screening for delirium to allow timely identification and appropriate treatment. Two recommended scoring systems for identification of delirium are the Intensive Care Delirium Scoring Checklist (ICDSC) and the Confusion Assessment Method for Intensive Care (CAM-ICU).²

## Objectives

To assess whether intensive care units within Scotland are screening for delirium on a daily basis and to test awareness of the current methods used for screening.

## Methods

This was a national survey of all 23 adult intensive care units in Scotland, via telephone over the course of one day. Nursing or medical staff in each unit, familiar with local policy were questioned to ensure validity of survey responses. Each unit was asked whether they screen for delirium on a daily basis and if they were aware of any screening tools used for delirium. Those units who screened for delirium daily were asked which screening methods they used (CAM-ICU or ICDSC) and whether nursing/medical staff or a combination carried this out primarily.

## Results

When asked 16 out of the 23 (69.57%) units in Scotland stated they screened for delirium on a daily basis.

Screening was done primarily by the nursing staff in 50% (8) of these units, with the other 25% by doctors and 25% both medical and nursing staff.

Of the 91.3% of units aware of recommended screening tools for delirium, all stated that they used the CAM-ICU screen.

## Conclusion

Daily screening for delirium using either CAM-ICU or ICDSC is the gold standard for early identification of a patient with delirium. Increased awareness of delirium through screening allows a prompt assessment of causes and a more sensitive holistic and pharmacological patient management plan to limit morbidity and mortality. In general, intensive care units in Scotland are screening for delirium daily and have a good awareness of the methods used to screen for delirium.

**Figure 1:**
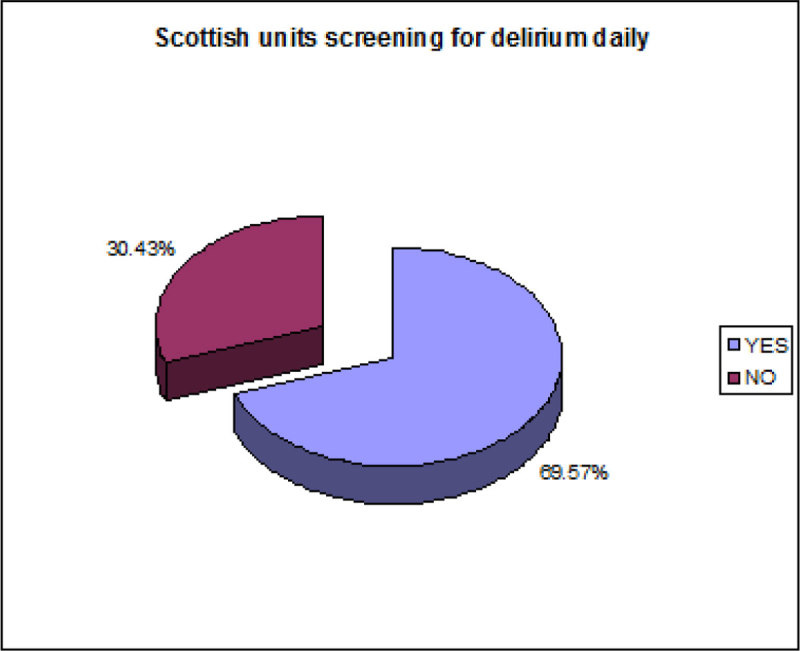
**Scottish units screening for delirium.**

**Table 1:** Who screens for delirium?

	Medical staff	Nursing Staff	Both
Number	4	8	4
Percentage	25%	50%	25%
